# Mitochondrial Phenotype as a Driver of the Racial Dichotomy in Obesity and Insulin Resistance

**DOI:** 10.3390/biomedicines10061456

**Published:** 2022-06-20

**Authors:** Filip Jevtovic, Polina M. Krassovskaia, Christian A. Lopez, Kelsey H. Fisher-Wellman, Ronald N. Cortright, Nicholas T. Broskey

**Affiliations:** 1 Human Performance Laboratory, Department of Kinesiology, East Carolina University, Greenville, NC 27858, USA; jevtovicf21@students.ecu.edu (F.J.); krassovskaiap18@students.ecu.edu (P.M.K.); lopezch19@students.ecu.edu (C.A.L.); cortrightr@ecu.edu (R.N.C.); 2 East Carolina Diabetes and Obesity Institute, East Carolina University, Greenville, NC 27858, USA; fisherwellmank17@ecu.edu; 3 Department of Physiology, Brody School of Medicine, East Carolina University, Greenville, NC 27858, USA

**Keywords:** insulin, African American, mitochondria, metabolic flexibility, skeletal muscle

## Abstract

African Americans (AA) are disproportionately burdened by metabolic diseases. While largely unexplored between Caucasian (C) and AA, differences in mitochondrial bioenergetics may provide crucial insight to mechanisms for increased susceptibility to metabolic diseases. AA display lower total energy expenditure and resting metabolic rate compared to C, but paradoxically have a higher amount of skeletal muscle mass, suggestive of inherent energetic efficiency differences between these races. Such adaptations would increase the chances of overnutrition in AA; however, these disparities would not explain the racial difference in insulin resistance (IR) in healthy subjects. Hallmarks associated with insulin resistance (IR), such as reduced mitochondrial oxidative capacity and metabolic inflexibility are present even in healthy AA without a metabolic disease. These adaptations might be influential of mitochondrial “substrate preference” and could play a role in disproportionate IR rates among races. A higher glycolytic flux and provision of shuttles transferring electrons from cytosol to mitochondrial matrix could be a contributing factor in development of IR via heightened reactive oxygen species (ROS) production. This review highlights the above concepts and provides suggestions for future studies that could help delineate molecular premises behind potential impairments in insulin signaling and metabolic disease susceptibility in AA.

## 1. Introduction

African Americans have disproportionately higher rates of obesity that persist across all ages and genders [1]. According to the Office of Minority Health, non-Hispanic African American (AA) adults are 1.3 times (47.9% vs. 37.4%) more likely to have obesity compared to non-Hispanic Caucasians (C) [1]. Likewise, AA children and adolescents are 1.4 times more likely to have obesity compared to C children and adolescents [1]. Furthermore, the obesity gap amplifies across gender with AA women being 1.5 times more likely to have obesity compared to C women. In fact, 55% of all AA women in the US can be classified in the obese body mass index (BMI > 30) category [2]. A contributing factor to these racial disparities is likely multifactorial, but several hypotheses have been proposed. One such hypothesis is that overall lower physical activity underlies these disparities, as 50.3% of AA do not meet the federal physical activity guidelines compared to 38.9% of their C counterparts [1]. Though the influence of socioeconomic status is not to be understated, it is worth noting that this pattern persists across the middle- and lower-income levels, and in males, this discrepancy grows even further at high-income levels [3]. Moreover, both AA and C races follow a similar pattern where the level of education is inversely related to the prevalence of obesity; however, rates of obesity are persistently higher among AA women at any education level [3]. Several studies have reported that differences in disease prevalence exist even after adjusting for socioeconomic status, access to healthcare, and quality of healthcare, suggesting that inherent physiologic differences exist and play a role in racial disparities in obesity and insulin resistance (IR) [4,5]. The purpose of this review is to provide a current state of the literature for mechanisms intrinsic to skeletal muscle metabolism in AA that may lead to increased metabolic disease risk. A particular emphasis is placed on skeletal muscle mitochondrial bioenergetics as a mediator of unraveling these inherent disparities and potentiating disproportionately higher risk of obesity and IR in AA.

## 2. Mitochondrial Efficiency Increases Predisposition of African Americans to Obesity

### 2.1. Energy Expenditure

The primary cause of obesity is often attributed to an energy imbalance from energy consumed and expended. Thus, it is essential to consider energy expenditure and its impact in the development of obesity as a causative factor in the racial disparity in the development of obesity. For example, it has been well documented that both total energy expenditure (TEE) and resting metabolic rate (RMR) are lower in AA compared to C counterparts across multiple age groups and regardless of gender or obesity status [6,7]. Partially responsible for the lower RMR is a lower mass of organs with high metabolic rates (i.e., liver, heart, etc.) in AA compared to C [8]. However, as these organs themselves do not comprise most of the active metabolic tissue in the body, it is essential to consider the role of skeletal muscle in TEE. Skeletal muscle itself comprises about 40% of total body weight and thus contributes substantially to energy expenditure as it is a prominent constituent of RMR [9]. Furthermore, considering that RMR comprises ~65% of TEE, skeletal muscle mass plays an essential role in determining TEE [9,10]. Paradoxically, while AA seem to have a greater percentage of relative skeletal muscle mass, they exhibit lower RMR making them more susceptible to overnutrition [7,11]. As skeletal muscle metabolism is largely dictated by mitochondrial bioenergetics, it is important to note the mitochondrial DNA variations that exist between AA and C populations and their effect on RMR, TEE, and overall risk for metabolic disease [12,13]. When compared to common European haplogroups (i.e., H, JT), common African haplogroups (i.e., L0, L2) exhibit significantly lower RMR and TEE even after adjusting for lean mass [14]. Furthermore, it is noteworthy to recognize that even within common racial haplogroups (i.e., AA), RMR and TEE can vary significantly [14]. Accordingly, future studies should attempt to control for these variances whenever possible, as these factors can be influential of the findings and introduce variability within a group. Taken together, these data provide evidence that certain genetic components may help drive racial dichotomies in energy metabolism, ultimately, making AA more susceptible to overnutrition.

### 2.2. Mitochondrial “Substrate Preference”

**Figure 1:**
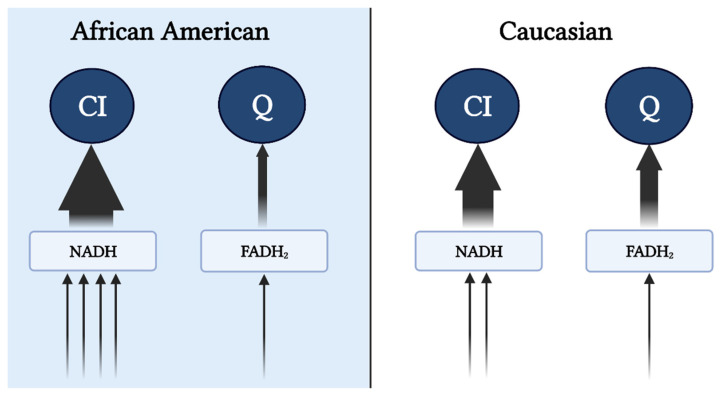
Preferential input of electrons into CI via NAD-linked substrates in AA. Heightened glycolytic flux ensues greater provision of NAD-linked pathways. From cytosol, NADH is brought into the mitochondrial matrix via malate-aspartate, G-3-P, and/or lactate shuttle(s) subsequently supporting the CI-linked respiration (described in the subsequent text). Supporting respiration through pyruvate, malate, and glutamate provides a 4:1 ratio (represented by the arrows) of NAD to FADH reduction with a complete TCA cycle. In contrast, Caucasians have a preferential respiration through succinate and fatty oxidation pathways resulting in a 2:1 reduction in NAD and FADH. This image was created using BioRender.com, accessed on 11 May 2022.

Reduced mitochondrial content and respiratory capacity have been reported in AA women [15,16], which is accompanied by lower maximal coupled and uncoupled respiration rates that persist despite adjustments for oxidative fiber-type content [15]. Additionally, lower state 3 (ADP stimulated), state U (uncoupled), and state 4 (leak) respiration have been observed in permeabilized fibers after normalization to mitochondrial content [16]. While it is tempting to ascribe lower maximal coupled and uncoupled respiration to mitochondrial dysfunction, it must be noted that oxidative phosphorylation (OXPHOS) and electron transfer capacity (independent of ATP synthase) under non-physiological conditions (lack of control over adenylate pool) provide limited information regarding mitochondrial bioenergetic function. Furthermore, the assays used in these studies were performed using mixed substrates during high-resolution respirometry, which could misrepresent these findings as mixed substrates prohibit the delineation of “substrate preference”. For example, the lower capacity (*J*O2) observed in AA could be largely due to the preferential utilization of NAD-linked, rather than FAD-linked substrates. The increase in glycolytic flux and decrease in fatty acid oxidation in AA compared to C (see subsequent sections) would increase the availability of NADH and NAD-linked substrates (i.e., pyruvate), potentiating preferential input of electrons to complex I (Figure 1), rather than Q pool (via CII or electron-transferring flavoproteins). In this case, more protons (~10 if entering at CI versus ~6 if entering at CII) are pumped out per reduced oxygen, resulting in a lower oxygen turnover (*J*O2) but greater efficiency (ATP/O ratio), leading to greater ATP production per amount of substrate utilized. This is in line with the evidence that AA have shorter recovery times during isometric exercise, consistent with a higher ATP synthesis rate, suggesting a greater energetic efficiency in AA [17]. This notion in concert with the lower recorded energy expenditure suggests a greater metabolic efficiency in AA compared to C.

**Figure 2:**
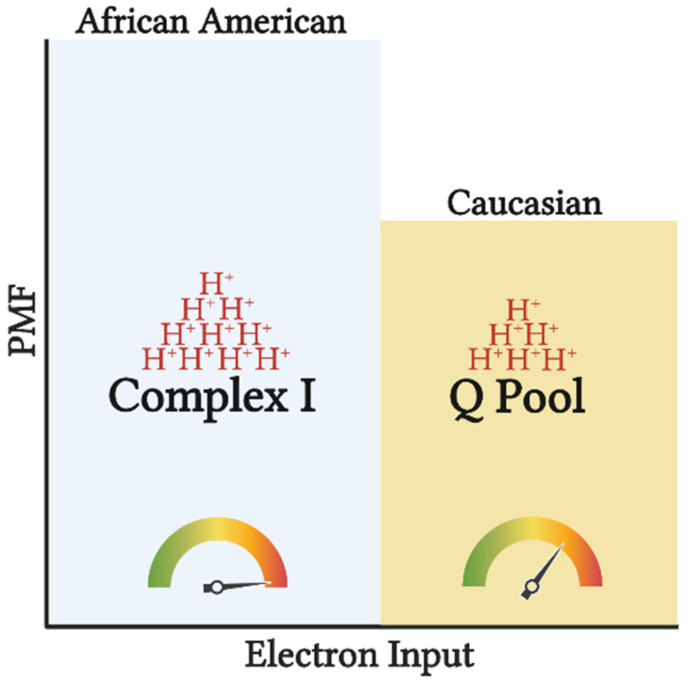
Tightly coupled mitochondrial respiration and NAD-linked substrate input potentiates greater efficiency (depicted by the gauge) in AA. Input of electrons into CI compared to the Q pool, will generate a greater H^+^ translocation across inner mitochondrial membrane (depicted by the H^+^ stacks) increasing the proton motive force (PMF), and subsequently ATP production. Such setup allows for greater ATP synthesis per substrate utilized, increasing the chances of overnutrition and subsequently obesity. This image was created using BioRender.com, accessed on 11 May 2022.

It is feasible that evolutionary adaptations have occurred as a result of tropical climates and lower accessibility to nutrients to maximize the efficiency of nutrient use and yield the highest ATP free energy [18]. Furthermore, along with the thrifty phenotype hypothesis, the AA mitochondrial phenotype would support maximal ATP and minimal heat production per calorie utilized [19]. This tight coupling of mitochondrial respiration to drive ATP synthesis would ensure greater efficiency in the utilization of carbon backbones provided by oxidized substrates. Additionally, high ETS efficiency and/or preferential use of NAD-linked substrates yields a more polarized mitochondrial membrane potential that is in inverse relation to the electron flow (Figure 2). Such a phenotype would explain the lower oxygen consumption rate and manifested as a lower RMR. This adaptation would likely be advantageous in scenarios of low and inconsistent nutrient availability; however, tightly coupled mitochondria in the presence of readily available nutrients would predispose AA individuals to overnutrition and promote disproportionate rates of obesity between races in an energy affluent environment. It must be noted that lower mitochondrial content and oxidative capacity are only in part influential on the amount of substrate being oxidized. Considering that the energetic state is derived from the cellular redox state, ATP production will take precedence over oxidative capacity in directing cellular bioenergetics or energy transfer from substrate to ATP production. Simply stated, the amount of oxidized substrate is dictated by the energy demand, rather than the energy input into the system. This suggests that regardless of the change in oxidative capacity contingent upon the shift in mitochondrial content, mitochondrial efficiency is a major determinant of substrate oxidation. While the chances of overnutrition increase considering the lower energy expenditure in AA, such an effect would not explain the racial difference in IR in healthy subjects [20]. Interestingly, mitochondrial capacity was inversely related to peripheral insulin sensitivity in work carried out by DeLany and colleagues, suggesting that this mitochondrial phenotype is a contributing factor to IR in AA [15]. Similarly, lower mitochondrial capacity has been reported in type 2 diabetics (T2D), inversely correlated with glycemic control, and directly corelated with metabolic inflexibility [21,22]. Accordingly, while in AA lower oxidative capacity might not stem from mitochondrial functional impairments, it can still have a role in increasing their predisposition to IR.

## 3. Mitochondrial Phenotype Increases Predisposition of African Americans to Insulin Resistance

### 3.1. Insulin Resistance and Metabolic (In)Flexibility

Skeletal muscle is responsible for ~80% of postprandial glucose uptake and yet, AA paradoxically display higher rates of IR despite having higher skeletal muscle mass [13]. Predictive measures of IR, such as disposition and β-cell function indices, are noted to be higher in AA [13]. Furthermore, in conditions of normal glucose tolerance, evidence of upregulated β-cell function as well as a rightward shift in the glucose allostasis model (relationship between insulin sensitivity and acute insulin response) is present [13]. Thus, the chronic hyperinsulinemia observed in AA must be considered as another factor in this racial disparity due to its strong association with obesity and other metabolic diseases (i.e., T2D) [14].

All factors considered, it seems that impaired glucose disposal at peripheral tissues lie at the core of racial disparities in glycemic control [13]. This paradigm observed in normoglycemic AA parallels a similar model of metabolic abnormalities that occur with IR in C [15]. Regarding IR, the progressive increase in insulin secretion in response to decreased peripheral tissue sensitivity leads to β-cell “stress” and subsequently exhaustion, demise, and dedifferentiation [16]. While IR is traditionally negative in terms of cardiometabolic disease with C, in the AA population, and in the context of inherent increased insulin output, it may serve as a protective mechanism against weight gain and obesity-related diseases (i.e., less fuel availability intracellularly for storage) [14]. However, genetically driven impairments in glucose uptake could predispose AA to a greater risk of T2D development as well and be further amplified in situations of overnutrition [14].

Metabolic flexibility has been implicated as a major predictor of insulin resistance and metabolic disease. Under conditions of energy metabolism unaffected by metabolic diseases, a metabolically *flexible* state can be characterized by mitochondria alternating between substrates based on physiologic needs and nutritional states. In contrast, in the state of metabolic *inflexibility*, crosstalk and regulation of substrate choice and substrate oxidation via metabolic and cell signaling events are dampened. Metabolic inflexibility, as observed in subjects with obesity and T2D, displays itself as a lack of substrate switching across changes in physiological states—a switch from the fasted state to a fed state and back [23].

Metabolic inflexibility and consequently the lack of substrate switching itself could have implications in the racial dichotomy between AA and C. Lower fat but higher carbohydrate oxidation and overall metabolic inflexibility have been reported in AA in the postabsorptive state and during high-fat and low-fat diets, euglycemic clamps, and epinephrine-induced lipolysis [24]. An inability to switch between substrates during eucaloric macronutrient-manipulated diets suggests that lean AA participants have an inherent preference for carbohydrate use. Moreover, it is worth noting that AA women display greater fasting plasma insulin during the low-fat and high-fat diet, which was contrary to C participants [24]. Additionally, contrary to C participants, with insulin administration during the euglycemic clamp, AA failed to suppress fatty acid oxidation. Similar outcomes were observed during the high-fat diet where there was no change in fat oxidation nor carbohydrate oxidation, suggesting that the fatty-acid-induced inhibition of glucose metabolism (i.e., Randle cycle) is not as prominent in AA [24]. Together, these findings in AA women with either normal or obese BMI suggest that there is preferential carbohydrate oxidation and that the manipulation of the physiologic environment via dietary habits is not sufficient to overcome these intrinsic biological metabolic differences.

**Figure 3:**
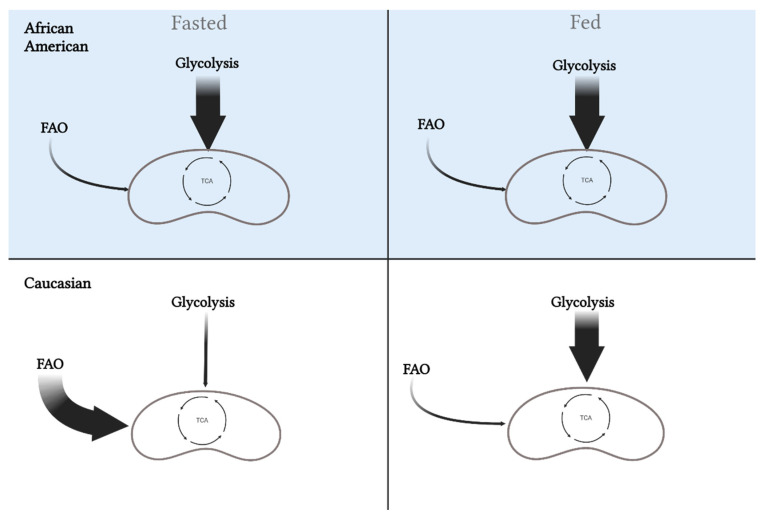
Differences in metabolic flexibility between African Americans and Caucasians. Metabolic flexibility is defined as the ability to switch between substrates (carbohydrates versus fat) in the fasted and fed state. African Americans rely primarily on glycolysis in the fasted state, which is maintained in the fed state. Alternatively, Caucasians will oxidize more fat in the fasted state and increase oxidization of carbohydrates in the fed state. These differences may play a role in the disparity for metabolic diseases between the races. This image was created using BioRender.com, accessed on 11 May 2022.

Metabolic inflexibility is influenced by the potential for lipid and carbohydrate oxidation determined by different muscle fiber-type compositions, and accordingly, variance in myocellular metabolic processes. Compared to C, AA display a lower proportion of insulin-sensitive type I muscle fibers geared towards oxidative metabolism combined with the higher proportion of type II glycolytic fibers, which results in a lower capacity for lipid oxidation at rest and during low-intensity exercise [24,25]. Furthermore, in normal weight and AA with obesity, these specific fiber-type distributions are associated with lower training adaptations, reduced oxidative capacity, and glycemic control [26,27,28,29,30,31]. Such fiber-type differences have been previously correlated with decreased insulin sensitivity and could influence the difference in obesity and T2D rates between these two races [27,31,32,33,34]. Specifically, higher glycolytic to oxidative muscle fiber-type ratio will potentiate higher glycolytic flux and could inhibit lipid oxidation via elevated production of malonyl CoA (i.e., reverse Randle cycle) [35]. Together, this altered substrate flux stemming from muscle fiber-type and innate metabolic differences potentiate metabolic inflexibility in AA (Figure 3).

### 3.2. Muscle Enzymatic Differences and Lower Fatty Acid Oxidation Potential

The previously described metabolic inflexibility of AA might be further influenced by phenotypic differences in metabolic pathways [26,29]. In muscle biopsies of the *vastus lateralis*, biochemical analyses showed lower oxidative and greater glycolytic pathway enzymatic activities in AA compared to C [29]. Sedentary AA males had a lower percentage of type I and a higher percentage of type IIa muscle fibers, which paralleled the higher phosphogenic and glycolytic enzyme activities when compared to C counterparts [29]. Additionally, as a surrogate measure of pathway predominance, Ama and colleagues reported a ~32% lower phosphofructokinase to oxoglutarate dehydrogenase ratio in C when compared to AA males. At the level of the TCA cycle, no differences were found in citrate synthase (CS), malate dehydrogenase, and oxoglutarate dehydrogenase between AA and C [29,35]. However, it is worth noting that CS was assessed in muscle from women with morbid obesity, which could be a reason for the lack of difference, considering that lower CS activity is associated with obesity [36]. Although CS is often a surrogate marker of mitochondrial content, observations of lower mitochondrial content in AA when compared to C would suggest an inherently lower CS content, but not CS activity per se [16,35].

While DeLany et al. observed lower succinate dehydrogenase in non-obese sedentary AA women when compared to their C counterparts [15], β-oxidation was not altered, as no differences in β-hydroxy acyl CoA were found [29,35]. Furthermore, lower mitochondrial and microsomal acyl-CoA synthetase activity was seen suggesting that decrements in oxidation stem from a lower fatty acid activation via acyl CoA synthetase [35]. This notion is supported by observed lower fatty acid oxidation in *rectus abdominus* strips and *vastus lateralis* muscle homogenates of AA compared to C [26,35]. In AA with normal and obese BMI classifications, there is lower oxidation of palmitate but not activated fatty acids (palmitoyl-CoA) [26]. Additionally, no differences in palmitate-carnitine were observed suggesting no difference at the level of carnitine-acylcarnitine translocase [26]. While Cortright et al. saw significant differences in subjects with obesity, the difference in palmitate oxidation between lean AA and C was only trending. This suggests that innate differences only predispose AA to decrements in fatty acid oxidation, and that significant effects might be dependent on and exacerbated with metabolic stress (i.e., overnutrition, obesity).

In accordance with the reverse Randle cycle theory, higher glucose uptake under conditions of innate hyperinsulinemia and higher glycolytic fiber-type content may be in part responsible for the downregulation of fatty acid oxidation [37]. Furthermore, lower succinate dehydrogenase would lead to a backup in the TCA cycle allowing for the accumulation and efflux of citrate into the cytosol. In skeletal muscle, this would lead to the accumulation of malonyl-CoA inhibiting CPT-1 and the rerouting of long-chain fatty acyl moieties towards esterification and storage [37]. The proposition of CPT-1 inhibition is unlikely based on the studies by Privette et al. [35] and Cortright et al. [26]; however, in these studies, muscle homogenate oxidation was measured in the presence of palmitate only, neglecting the potential effect of higher glucose flux. Additionally, the measures obtained do not mimic the postprandial state in which glucose availability and influx into the cell is highest and holds the greatest potential to influence substrate preference. The influence of glucose and dietary glycemic load on fat metabolism in AA has been highlighted in a recent review by Gower and Fowler [20]. The authors suggest that individuals with high postprandial insulin have a chronic “brake” on lipid metabolism predisposing them to obesity. Accordingly, AA compared to C lost significantly more fat mass on a low glycemic compared to low-fat diet. Such evidence would suggest that the combination of genetic hyperinsulinemia and high glucose availability potentiate glycolytic flux and lipid backup considering that fat and carbohydrate oxidation are mutually inhibitory. Lastly, more efficient OXPHOS and lower FAO increases the susceptibility of fatty acid accumulation with overnutrition. Together, such evidence puts fatty acid accumulation in the “spotlight” when it comes to the impairments in insulin sensitivity; however, this might not be the case when it comes to AA.

### 3.3. Ectopic Lipid Accumulation Is Not a Predictor of Insulin Resistance in AA

Lower fatty acid oxidation in parallel with metabolic inflexibility facilitates the partitioning of lipids towards ectopic accumulation. Excessive total and ectopic fat deposition are known to contribute to the development of IR. Interestingly, while patterns of ectopic fat deposition differ between AA and C, their contribution to IR is not clearly demonstrated. AA have a trend towards higher intermuscular adipose tissue and this difference becomes significant with increased adiposity; however, it is not a strong predictor of insulin sensitivity in AA [38,39,40,41]. Additionally, a similar intramyocellular lipid content has been observed between AA and C in a recent meta-analysis across all BMIs, which suggests that the total accumulation of the intramyocellular lipids is not a major contributor to differences in peripheral IR between the races [42]. Nonetheless, while intramyocellular lipid content is correlated with insulin sensitivity in C, there is no association between these parameters in healthy and overweight AA subjects, suggesting that other fat depots or specific lipid species take precedent in influencing insulin sensitivity in AA [43,44]. Compared to C, it has been shown that AA have significantly lower visceral adiposity but significantly greater subcutaneous adipose depots [42,45]. Furthermore, both visceral and subcutaneous adipose tissue have been correlated with IR in both healthy and glucose-intolerant AA subjects [46]. These observations suggest that peripheral IR in AA might be subject to the composition of specific muscular lipid species rather than quantities considering that the associations between ectopic lipid accumulation and insulin sensitivity in either healthy or AA with obesity are weak.

Intramyocellular lipids are composed of predominantly triacylglycerols, but also include diacylglycerols, ceramides, and long-chain fatty acyl-CoAs. Of these lipid species, ceramides and diacylglycerols have been implicated as drivers of lipotoxicity-induced IR [43]. While there is a lack of research regarding intramyocellular lipid profiling, plasma levels of C16:0 ceramides are shown to be similar between healthy AA and C [47]. Moreover, AA had trends towards higher C18:0, C18:1, C22:0, and C24:0 levels [47]. This trend would imply higher C22:0/C16:0 and C24:0/C16:0 ratios which have been associated with an inverse relationship with all-cause mortality and coronary heart disease [47,48]. Similarly, AA subjects have a better ceramide ratio (C18:1/C18:0)/(C18:1/C16:0), which has been implicated as an independent marker predictive of T2D even after adjusting for BMI, fasting glucose, and HbA1c [47,49]. In addition, while a trend towards overall higher total ceramide content was observed in healthy AA, the opposite was found in AA with metabolic disease potentially due to the lack of adjustment for any of the reported confounding variables (i.e., medication). Finally, the only significant difference between healthy groups was higher levels of sphingosine 1-phosphate (S1P) in AA [47]. However, contradictory findings regarding the involvement of S1P in IR exist making it hard to conclude if there is an association between insulin sensitivity and these lipid species in AA [44]. In the study by Jones Buie et al. [47], S1P levels were measured in blood and thus might not be representative of the skeletal muscle environment.

Collectively, these findings of the abovementioned studies make it hard to conclude that intracellular lipid accumulation is a predominate variable responsible for reduced insulin sensitivity in AA. Considering that lipid accumulation as well as metabolic flexibility are heavily influenced by mitochondrial function, the potential role of these and other organelles in IR should be further investigated. Particularly, the involvement of mitochondria in redox balance should be explored, considering the involvement of oxidative stress in the development of IR [50].

## 4. Oxidative Stress and Insulin Resistance

Reactive oxygen species (ROS) is a valuable signaling mechanism that potentiates many positive metabolic adaptations; however, chronically heightened oxidative stress could play a significant role in inducing IR. Chronically heightened ROS production is linked to IR via a more positive redox potential derived from higher H_2_O_2_ emission [50]. Elegantly described by Fisher-Wellman and Neufer [50], oxidative stress induces a global shift in the redox environment to a more oxidized state promoting the activation of stress-sensitive Ser/Thr kinases (i.e., JNK), which in turn leads to subsequent Ser/Thr phosphorylation of IRS1 and downstream signaling proteins to limit insulin sensitivity.

The notion of heightened oxidative stress and lower antioxidant capacity (lower glutathione and superoxide dismutase activity and expression) have been observed in AA both in vivo and in vitro regardless of obesity [51,52]. The heightened efficiency of the ETS potentiates a higher mitochondrial membrane potential and with tight coupling to ATP synthase instils high cellular energetic levels (ATP:ADP ratio). In an elevated redox state, “backpressure” is generated and applied on electrons attempting to enter the ETS and any excess of reducing equivalents is mitigated via electron leak [50]. At rest, electron leak is particularly heightened as the system is in its most reduced state [50]. Furthermore, in this state, the emission of superoxides will be highly influenced by changes to the membrane potential [53,54]. Accordingly, an oversupply (i.e., overnutrition) of electrons to the ETS will increase the chance of electron leak and promote free radical formation. Considering the higher mitochondrial efficiency and lower RMR and TEE in AAs, this population would be more susceptible to overnutrition-derived increases in ROS production. While the influence of overnutrition is associated with the development of IR, it does not explain the higher susceptibility seen in healthy (no history of a metabolic disease) AA. Thus, other mechanisms that could contribute to the heightened ROS production should be explored. In AA, we postulate that the increase in ROS production may stem from differential mitochondrial substrate oxidation and together with lower antioxidant capacity leads to a higher propensity to IR. While such mechanisms have not been directly tested, we will provide a theoretical premise behind possible means that could augment ROS production.

### 4.1. Relative Oversupply of Fatty Acids

The uptake, accumulation, and potential for lipid oxidation varies across muscle fiber types with slow-twitch muscle fibers having the highest potential for lipid oxidation [55]. In AA, independent of caloric surplus, there is a potential for a relative oversupply of fatty acids to oxidative fibers contingent upon the inadequacy of glycolytic fibers to oxidize fatty acids. Lower clearance of fatty acids in glycolytic muscle fibers creates a surplus and potentiates a shunt of fatty acids towards oxidative fibers in a process analogous to the cell–cell lactate shuttle. However, the predominantly higher glycolytic to oxidative muscle fiber ratio seen in AA could result in substrate oversaturation of oxidative fibers contributing to the fiber-type-specific lipotoxicity as well as ROS production [50]. While evidence regarding this mechanism is scarce, the combination of lower oxidative capacity and shifted ratio of glycolytic to oxidative muscle fibers could contribute to muscle lipid accumulation and subsequently lower insulin sensitivity.

An oversupply of fatty acids has been shown to generate higher ROS production. Fatty-acid-induced ROS production calls for the oversaturation of electrons at the Q pool via β-oxidation and FAD-linked substrates [50]. Considering that the entrance of electrons to the Q pool via CII is not constrained by membrane potential, an increase in β-oxidation (due to higher fatty acid availability) would allow for a further reduction in the Q pool [50]. In such a reduced state, the Q pool has limited ability to accept electrons from CI and could result in the reverse flow of electrons to CI. Furthermore, the reduction of the Q pool will increase the reducing equivalents “pressure head” toward CIII and elicit CIII-specific electron leak. Together, these mechanisms account (at least in part) for the increase in superoxide production [50]. Potential for this mechanism to be in part responsible for heightened ROS production in AA would be dependent on the relative fatty acid oversupply to oxidative muscle fibers. However, this hypothesis needs to be tested before any association can be assumed.

### 4.2. Glycolytic Flux and ROS Production

Considering the predominance of glycolytic flux in AA, it would be reasonable to postulate that there is a preeminent provision of glycerol-3-phosphate (G-3-P), malate-aspartate shuttles (MAS), and lactate shuttle (LS) to transfer the electrons produced in glycolysis to the ETS.

While no significant difference in maximal ADP-stimulated respiration via NAD-linked substrates has been seen between muscle fibers using high resolution respirometry, some difference persists in the respiration capacity with G-3-P. Moreover, while the G-3-P shuttle is not exclusive to glycolytic fibers, there is a four- to ten-fold higher respiration in the presence of G-3-P when compared to oxidative muscle fibers, which is consistent with a higher dependence on glycolysis [56]. The G-3-P shuttle allows for cytosolic reducing equivalents to enter directly into the Q pool via mitochondrial G-3-P dehydrogenase (mGPDH), independent of the membrane potential [57]. This mechanism of G-3-P-derived ROS production can be seen as analogous to the Q pool reduction via beta-oxidation-derived FADH (entering electron-transferring flavoproteins (ETF)). Considering that electron input to the Q pool in either case (via mGPDH or ETF) is not constricted via the membrane potential, reduction in the Q pool would be largely dependent on G-3-P availability. Accordingly, a higher glycolytic flux would potentiate the oversaturation of the Q pool via mGPDH leading to a reverse flow of electrons to CI or CII, and/or an increase in the reducing equivalents “pressure head” at CIII, or even at the level of mGPDH itself [57]. Finally, it is worth noting that G-3-P-dependent superoxide production from succinate dehydrogenase is heightened at low concentrations of succinate and high G-3-P concentrations [58]. While the complete absence of succinate is not probable, lower succinate dehydrogenase, as observed in AA, would ensue lower succinate concentrations within mitochondrial matrix [29]. Thus, within the mitochondrial matrix, this would allow for greater electron leak at the site of succinate dehydrogenase [57]. Heightened glycolytic flux and predominance of glycolytic fibers would suggest that this mechanism could play a role in heightened ROS production in AA, but this remains to be tested.

To mitigate the high redox state and oxidize cytosolic NADH, other shuttles such as the MAS and LS might be active. Via the MAS, electrons from cytosolic NADH are brought to the mitochondrial matrix, where they are fed directly to the CI. Provision of the MAS maximizes NAD reduction and minimizes FADH2 production at the level of TCA cycle. It must be noted that entrance of the cytosolic NADH-derived electrons into the mitochondrial matrix via MAS is driven by the proton motive force and enhanced by the increasing membrane potential. Accordingly, high coupling efficiency would potentiate the increase in membrane potential and support the MAS in AA. In terms of ROS production in muscle, higher MAS provision and oxidation of MAS-supporting substrates (glutamate and malate) results in a substantial increase in H_2_O_2_ emission at the level of CI, CIII, and alpha-ketoglutarate [59]. Additionally, in permeabilized muscle fibers, a continual source of ROS emission has been observed at the level of pyruvate dehydrogenase complexes [60]. This emission of ROS was further observed to be exacerbated with low glutathione levels and dependent on pyruvate availability. Accordingly, under states of heightened glycolytic flux and low levels of glutathione, as is seen in AA, ROS emission would be significantly increased and lead to near constant H_2_O_2_ emission. While this mechanism would be in synchrony with high mitochondrial efficiency and previously described metabolic characteristics, the support of MAS in skeletal muscle is low. However, in AA, the specific adaptations that increase the provision of MAS remain unexplored.

As a major producer of lactic acid, the LS is highly active in both anaerobic and aerobic conditions within the skeletal muscle [61]. Considering that the interconversion of pyruvate to lactate via lactate dehydrogenase is coupled with the oxidation of NADH, the LS serves to bring cytosolic NADH to the mitochondria. In muscle, mitochondrial basal respiration and coupling with lactate has been seen to closely resemble that of pyruvate [62]. On the other hand, higher mitochondrial respiration and similar coupling with lactate were observed even when equal amounts of lactate and pyruvate are supplied to isolated mitochondria, but this is a non-physiological state considering that the amount of lactate exceeds that of pyruvate in vivo [63]. Interestingly, muscle mitochondrial respiration with lactate shows a trend towards higher H_2_O_2_ production at the level of lactate-targeting enzymes [61,62]. Additionally, considering that lactate entering the mitochondria will be oxidized to pyruvate, the provision of LS could have an implication in ROS production at the site of mitochondrial lactate-targeting enzymes and PDH; however, it remains unexplored in AA.

**Figure 4:**
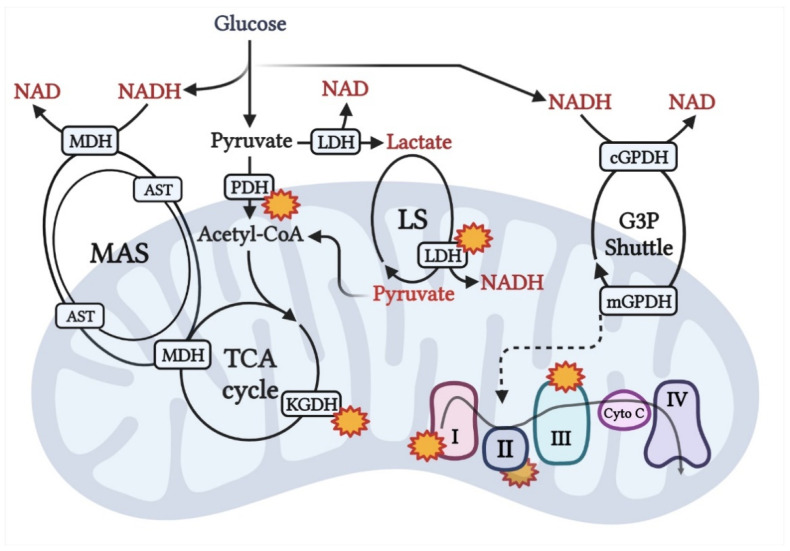
Transport of cytosolic NADH into the mitochondrial matrix via malate-aspartate, lactate, and G-3-P shuttle(s) and potential sites or ROS emission (represented by the star symbol). For further explanations see Section 4.2. Abbreviations: MDH, malate dehydrogenase; PDH, pyruvate dehydrogenase; AST, aspartate transaminase; KGDH, α-ketoglutarate dehydrogenase; LDH, lactate dehydrogenase. This image was created using BioRender.com, accessed on 11 May 2022.

Overall, the state of higher oxidative stress in AA could be compounded by an imbalance between ROS production and scavenging. Heightened potential for ROS production at the described sites (Figure 4) and lower glutathione and lower superoxide dismutase activity and expression could be a factor in IR observed in healthy AA; however, these propositions remain to be tested.

## 5. Association of Mitochondrial Dynamics with Insulin Resistance in AA

AA display altered gene expression and protein content responsible for mitochondrial membrane transport, fission, and fusion, which may contribute to the differences in mitochondrial bioenergetics between races [64]. Transacylase Tafazzin (TAZ), a mitochondrial membrane transport protein, plays a key role in alleviating high-fat diet-induced insulin resistance, inflammation, and palmitate-induced impairments in insulin signaling [65]. Interestingly, AA women have lower TAZ gene expression in skeletal muscle, which could contribute to the disparities in mitochondrial function seen between races. Furthermore, lower TAZ expression in AA correlates with lower mitochondrial respiration suggesting a potential role of TAZ in regulating mitochondrial function that differs between races.

In addition to skeletal muscle TAZ, lower expression of TIMM8A and TIMM17B (mitochondrial import proteins) in AA women supports a lower capacity for mitochondrial fission and fusion as well as a potential for altered overall mitochondrial functioning [64]. TOMM34 is also lower in AA and plays a role in the regulation of heat shock proteins [66] which are associated with insulin resistance [67,68,69]. Heat shock proteins allow for the inhibition of c-Jun amino terminal kinase (JNK) phosphorylation which subsequently reduces the inhibition of IRS-1 phosphorylation and PI3K-AKT signaling [68,70,71]. Additionally, it has been shown that the downregulation of JNK will divert glucose from oxidation and promote non-esterified fatty acid oxidation in myotubes [72]. This suggests that alterations in heat shock proteins will lower the ability to reduce JNK and may result in lower fatty acid oxidation and subsequent non-esterified fatty acid accretion [72,73]. Finally, JNK activation and interplay with Sab, an outer membrane mitochondrial protein, has been associated with the greater production of reactive oxygen species through the impairment of mitochondrial respiration [74,75,76]. Thus, cellular stressors (i.e., pro-inflammatory cytokines, free fatty acids) can activate JNK and with Sab-mediated inhibition of mitochondrial ETS, resulting in a JNK activation loop (JNK-Sab-mitochondria-ROS). This creates a vicious cycle resulting in the sustained activation of JNK and production of ROS which would subsequently promote insulin resistance [74,75,76,77].

Moreover, it is important to note that skeletal muscle content of FIS1 and MFN2 has also been shown to be lower in AA when compared to C [64]. Dube et al. [64] reported significantly higher OPA1 in the skeletal muscle of AA contributing to this difference in mitochondrial dynamics between races. Considering the inverse relationship between mitochondrial function and insulin resistance, an offset in the balance of the mitochondrial proteins (i.e., MFN1 and MFN2, FIS1, OPA1) will influence the fission–fusion dynamics and can be considered as a precursor for mitochondrial-derived alterations in insulin sensitivity [64,78]. This notion is supported by the positive correlation of MFN2 with peripheral insulin sensitivity [64,78]. Taken together, an imbalance between mitochondrial fission and fusion pathways appoints mitochondria as a critical factor influencing racial dichotomies in IR.

## 6. Other Factors Influencing Obesity and Insulin Resistance in African Americans

### 6.1. Uncouplers

Considering the importance of mitochondria in fuel partitioning and oxidation, it is worth noting the differences in uncoupling proteins observed between races and the influence of these on substrate preference and energy expenditure. Uncoupling proteins (UCP) are a class of mitochondrial inner-membrane carrier proteins that dissipate the electron transport system (ETS)-generated proton gradient thereby uncoupling oxidative phosphorylation. Accordingly, these proton transporters reduce the amount of ATP synthesis per amount of the oxidized substrate, subsequently increasing the metabolic rate. A significant association exists between the C allele in the UCP3 exon 5 variant and lower REE in AA women but not in C counterparts [79]. Two polymorphisms (V102I and exon 6-splice) of UCP3 have been detected in individuals with early onset obesity and T2D and are exclusive to AA [79]. Additionally, subjects heterozygous for the exon 6-splice have reduced basal fat oxidation rates and markedly higher respiratory quotient (RQ) [79]. This suggests that this alteration in UCP3 function influences fuel partitioning through the efflux of fatty acids out of the mitochondrial matrix and subsequently decreases the availability of fatty acids for oxidation [79]. However, contrary to Argyropoulos et al. [79], a lack of association of UCP3 exon 6-splice polymorphism and RQ was reported by Chung et al. [80]. UCP3 is most abundant in glycolytic muscle fibers with low oxidative capacity suggesting that an efflux of fatty acids may be protective against the accumulation of fatty acids in the mitochondrial matrix, subsequently inferring a greater dependence on glycolytic substrates [81]. In addition to UCP3, UCP2 is expressed in various tissues including skeletal muscle and in part serves to uncouple respiration which subsequently affects energy expenditure [82]. While an association between UCP2 exon 8 polymorphism and obesity measures has been seen in the study by Yanovski et al. [83], there were no significant differences between races and no effect of polymorphism on energy expenditure. Although there is evidence of uncoupling proteins influencing fuel partitioning and energy expenditure, considering the limited data and the lack of consistency between findings, further studies are needed to delineate the existence of racial differences that could influence fatty acid handling and subsequently IR.

### 6.2. Peroxisomes

With decrements in mitochondrial fatty acid oxidation, it is reasonable to assume a divergent flow of long-chain fatty acids to peroxisomes. Peroxisomes are ubiquitously expressed organelles which alongside mitochondria are a major site of β-oxidation; however, peroxisomal fatty acid oxidation is not linked to energy production. Oxidation within these organelles allows for the shortening of long and very-long-chain fatty acids yielding acyl-carnitine metabolites that enter the mitochondria independent of mitochondrial fatty acid transport systems (i.e., ACS, CPT1). Peroxisomes are responsive to environmental stimuli and the upregulation of peroxisomes in human skeletal muscle, as well as primary myotubes, with lipid overload (i.e., high-fat diets) has been observed [84]. Accordingly, peroxisomal lipid oxidation correlates with both intramyocellular lipid content and inhibition of CPT-1 [84]. In AA, the route of peroxisome-derived medium-chain acyl-CoA bypasses two potentially rate-limiting steps (ACS and CPT-1) in fatty acid oxidation in AA; thus, the provision of this pathway is highly likely. Partial β-oxidation within peroxisomes is governed by a class of reactive oxygen species (ROS)-producing enzymes collectively termed acyl-CoA oxidases (i.e., palmitoyl-CoA oxidase). Respiratory reactions in peroxisomes, including partial β-oxidation, results in oxidation products and the production of hydrogen peroxide [85]. To mitigate the generation of ROS (i.e., H_2_O_2_), peroxisomes have a well-developed antioxidant system. The interplay between pro- and antioxidant systems within these organelles establishes a redox balance that is dependent on both the production and scavenging of ROS and further influenced by the physiological state (i.e., skeletal muscle lipid accumulation) [84,86]. The antioxidant system within peroxisomes contains various ROS-metabolizing enzymes (i.e., catalase, superoxide dismutase 1, etc.) [87]. Additionally, free radicals can be scavenged non-enzymatically by antioxidants such as glutathione. While antioxidant systems in the skeletal muscle of AA have not yet been explored, limited data exist from plasma and umbilical vein endothelial cell studies. These studies report that AA have lower plasma levels of glutathione with trends of more oxidized glutathione, as well as lower superoxide dismutase 1 cellular activity and superoxide dismutase 2 protein expression [51,52]. Though data regarding peroxisomal involvement in the redox biology of skeletal muscle are limited, the data may be significant for AA considering the reports of higher oxidative stress, greater intramuscular adipose tissue, and decreased ability for mitochondrial β-oxidation.

## 7. Conclusions

Considering the complex nature and lack of understanding of the cellular processes, future researchers should strive to elucidate molecular mechanisms governing peripheral IR in AA. Furthermore, considering that the association of intramyocellular lipid accumulation and IR is weak even after numerous in vivo studies, research should turn towards ex vivo studies that could help delineate molecular premises behind potential impairments in insulin signaling. Finally, considering that very few authors have investigated the relationship and influence of mitochondrial outcomes on IR in AA, more should be carried out to assess and establish the model of mitochondrial bioenergetics in this population. Specifically, the areas of mitophagy and mitochondrial calcium homeostasis have been implicated in the development of metabolic disease yet remain largely unexplored across different races [88,89,90]. The focus on mitochondrial bioenergetics will also help elucidate the inherent lower energy expenditure in AA vs. C and could provide future targets to prevent obesity and T2D in this population. Collectively, these recent findings represent a new frontier in mitochondrial bioenergetics between races.
